# Prophase roles of replication protein A in crossover formation and meiotic progression

**DOI:** 10.71150/jm.2604001

**Published:** 2026-06-18

**Authors:** Rose M. Lee, Keun Pil Kim, Jeong H. Joo

**Affiliations:** Department of Life Sciences, Chung-Ang University, Seoul 06974, Republic of Korea

**Keywords:** meiotic recombination, RPA, crossover, prophase I

## Abstract

Meiotic recombination is initiated by programmed DNA double-strand breaks (DSBs), which are subsequently processed to generate single-stranded DNA (ssDNA). Replication protein A (RPA), a heterotrimeric ssDNA-binding complex, plays essential roles in DNA replication, repair, and recombination; however, the specific functions of RPA in meiotic recombination progression and chromosome morphogenesis remain unclear. Here, we investigate the role of RPA in recombination and meiotic progression by conditionally depleting Rfa1, the large subunit of the RPA complex, using an auxin-inducible degron (AID) system in *Saccharomyces cerevisiae*. We show that Rfa1 depletion causes severe defects in meiotic recombination, including impaired DSB processing, defective chromosome axis assembly, compromised synaptonemal complex formation, and failure of ZMM-dependent crossover recombination. Notably, inhibition of Mek1 protein kinase activity, which bypasses the recombination checkpoint, does not rescue these defects in Rfa1-depleted cells. Together, these findings identify RPA as a key factor that stabilizes recombination intermediates and coordinates prophase I events with chromosome synapsis and crossover formation during meiosis.

## Introduction

Meiotic recombination is a tightly regulated process that begins with the formation of programmed DNA double-strand breaks (DSBs), which are subsequently repaired to generate crossovers (COs) between homologous chromosomes (Bishop and Zickler, 2004; Zickler and Kleckner, 1999). These COs provide physical connections between homologs, ensuring accurate segregation during meiosis I. DSB formation is catalyzed by the topoisomerase-like protein Spo11, which covalently binds to DNA ends and initiates homologous recombination (Keeney, 2001; Neale et al., 2005). In budding yeast, Spo11-bound short oligonucleotides are removed by the Mre11/Rad50/Xrs2 (MRX) complex and Sae2, generating substrates that are competent for extensive end resection (Lee et al., 2020; Neale et al., 2005; Paull and Gellert, 1998; Tamai et al., 2024; Wang et al., 2017). Subsequently, 5′–3′ exonuclease 1 (Exo1), the helicase–topoisomerase Sgs1/Top3/Rmi1 (STR) complex, and Dna2 extensively resect DSB ends to generate long-range 3′ single-stranded DNA (ssDNA) (Garcia et al., 2011; Mimitou and Symington, 2008; Zakharyevich et al., 2010; Zhu et al., 2008). The exposed ssDNA is rapidly coated by replication protein A (RPA), which stabilizes and protects the DNA from nuclease degradation and spontaneous DNA hairpin formation (Cannavo et al., 2013; Chen et al., 2013). RPA-coated ssDNA is then remodeled by the recombinases Rad51 and Dmc1, orthologs of bacterial RecA (Lao et al., 2013; Sung, 1997), which assemble into ATP-dependent nucleoprotein filaments on ssDNA that promote homology search and strand invasion into an intact homologous chromosome (Lao et al., 2013; Sung, 1994). This process leads to the formation of a displacement loop (D-loop) and a single-end invasion (SEI) intermediate, in which the invading 3′ ssDNA end primes DNA synthesis using the homolog as a template (Hunter and Kleckner, 2001; San Filippo et al., 2008). DNA synthesis then extends the invading ends and promotes the formation of double Holliday junctions (dHJs), with the second DSB end captured in a reaction facilitated by Rad52 and RPA (Hong et al., 2019b; Joo et al., 2024; Lao et al., 2008). These recombination intermediates are eventually processed by several nucleases and helicase complexes, Mlh1–Mlh3, Exo1, the STR complex, Slx1–Slx4, Mus81–Mms4, and Yen1, to produce both CO and noncrossover (NCO) products (Choi and Chung, 2026; De Muyt et al., 2012; Jessop and Lichten, 2008; Oh et al., 2007; Sanchez et al., 2021; Zakharyevich et al., 2010).

Mek1 is a meiosis-specific serine/threonine protein kinase in budding yeast that is activated in response to programmed DSB formation. Activated Mek1 phosphorylates Rad54, thereby inhibiting Rad51–Rad54 complex formation and suppressing Rad51-mediated recombination (Hollingsworth and Gaglione, 2019; Hong et al., 2013; Kim et al., 2010; Niu et al., 2005, 2009). In addition, the meiosis-specific protein Hed1 binds to Rad51 and further interferes with the Rad51–Rad54 interaction, thereby reinforcing suppression of Rad51 activity (Busygina et al., 2008; Crickard et al., 2018). Through these mechanisms, Mek1-dependent regulation suppresses DSB repair via sister chromatids and promotes the preferential use of homologous chromosomes as a repair template (Joo et al., 2022; Latypov et al., 2010; Niu et al., 2005, 2007; Terentyev et al., 2010). This meiosis-specific recombination pathway maintains homolog bias during the transition from interhomolog (IH)-SEIs to IH-dHJs, thereby promoting CO formation (Latypov et al., 2010; Niu et al., 2005, 2007; Terentyev et al., 2010). In the absence of Mek1 kinase activity, meiotic DSB repair shifts to a Rad51-dependent, sister-bias pathway that resembles the mitotic recombination pathway, in which the sister chromatid is preferentially used as the repair template (Hollingsworth and Gaglione, 2019; Latypov et al., 2010; Niu et al., 2005, 2007, 2009; Terentyev et al., 2010).

RPA is a conserved heterotrimeric ssDNA-binding protein complex that functions in multiple DNA metabolic pathways, including DNA replication, repair, and recombination (Brill and Stillman, 1991; Chen and Wold, 2014; San Filippo et al., 2008; Symington et al., 2014; Wold, 1997). In *Saccharomyces cerevisiae*, RPA is composed of Rfa1, Rfa2, and Rfa3 (corresponding to RPA70, RPA32, and RPA14 in humans). During recombination, RPA rapidly associates with ssDNA tails, safeguarding it from nucleolytic degradation and preventing the formation of inhibitory secondary structures (Brill and Stillman, 1991; Chen and Wold, 2014; San Filippo et al., 2008; Symington et al., 2014; Wold, 1997). RPA then facilitates the recruitment of the recombinases Rad51 and Dmc1, which assemble into nucleoprotein filaments that promote strand invasion and D-loop formation (Chen et al., 2008; Conway et al., 2004; Crickard and Greene, 2018; Hinch et al., 2020; Joo et al., 2024; Kowalczykowski, 2015; Morrical, 2015; Sheridan et al., 2008). Despite extensive characterization of RPA in DNA replication and mitotic homologous recombination (Brill and Stillman, 1991; Chen and Wold, 2014; San Filippo et al., 2008; Symington et al., 2014; Wold, 1997), the specific roles of RPA in meiotic chromosomes remain less clearly defined. Recent studies have shown that depletion of the RPA large subunit, Rfa1, results in defects in homolog bias during recombination in yeast meiosis (Chen et al., 2013; Joo et al., 2024; Sampathkumar et al., 2024).

**Fig. 1.**
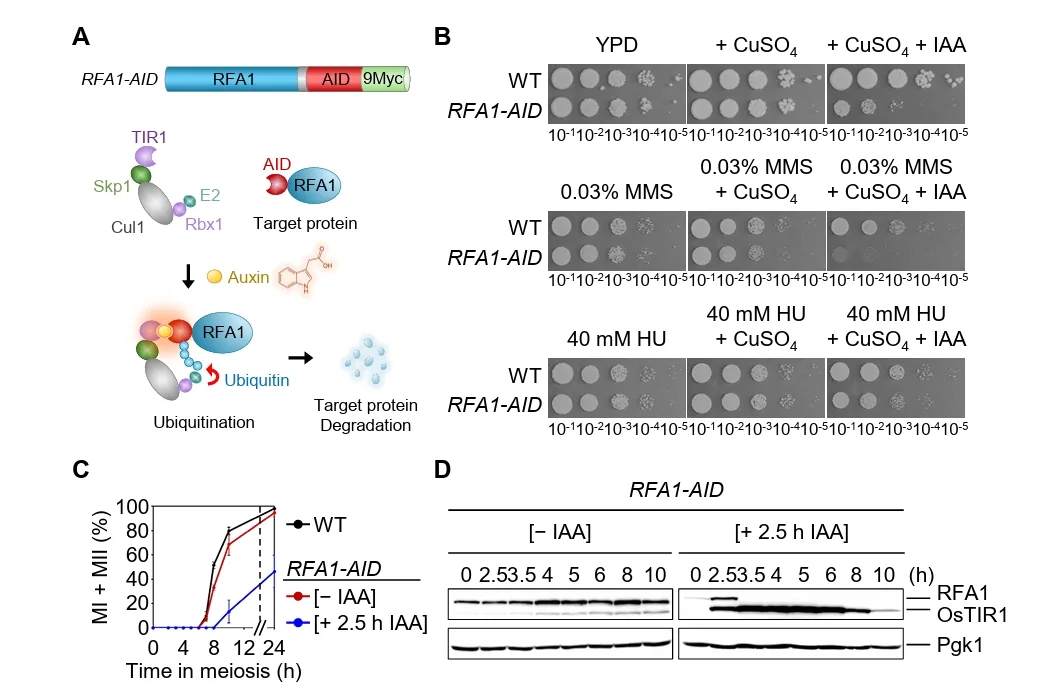
Conditional Rfa1 degradation via auxin-inducible degron alters DNA damage sensitivity and meiotic progression. (A) Schematic representation of the *RFA1*–*AID* construct and the auxin-inducible degron (AID) system. Upon auxin (IAA) treatment, *RFA1*–*AID* is ubiquitinated by the SCF^TIR1^ complex and subsequently degraded by the proteasome. (B) DNA damage sensitivity assay of wild-type (WT) and *RFA1*–*AID* strains. The 10-fold serial dilutions (from 10^-1^ to 10^-5^) were spotted onto YPD plates containing the DNA-damaging agents: 0.03% MMS, 40 mM HU, 20 μM CuSO_4_, or 2 mM IAA. Plates were incubated at 30°C for 2 days. (C) Meiotic nuclear divisions in WT and *RFA1*–*AID* strains. Data are presented as the mean ± standard deviation (SD) (N > 200/time point; three independent biological replicates). (D) Protein expression levels of Rfa1 and Pgk1 during meiosis in the *RFA1*–*AID* strain. CuSO_4_ (20 μM) was added at 2 h to induce OsTIR1 expression, followed by IAA (2 mM) treatment at 2.5 h to induce Rfa1 degradation. Proteins were detected using anti-Myc (for *RFA1*–*AID*–*9Myc*) and anti-Pgk1 antibodies. Unprocessed blot images are shown in Fig. S1B.

To investigate the roles of RPA during meiotic prophase, we examined its contribution to meiotic chromosome architecture and recombination progression, focusing on chromosome and recombinase dynamics. Since RPA is essential for cell viability, we employed a plant-derived auxin-inducible degron (AID) system to deplete the RPA large subunit, Rfa1, rapidly and specifically during meiosis (Morawska and Ulrich, 2013; Nishimura et al., 2009). In this system, addition of auxin promotes interaction between the AID-tagged protein and the TIR1 ubiquitin ligase complex, leading to ubiquitination and rapid proteasome-mediated degradation of the target protein (Fig. 1A; Morawska and Ulrich, 2013; Zimmerman et al., 2010). The rapid and conditional nature of this system enables functional analysis of essential proteins during meiosis. Using this approach, we demonstrate that depletion of Rfa1 disrupts the progression of recombination intermediates, chromosome axis formation, and synaptonemal complex (SC) assembly, ultimately impairing ZMM-dependent crossover designation. Here we propose that RPA plays a critical role in coordinating crossover recombination with meiotic chromosome morphogenesis.

## Materials and Methods

### Strains

All strains were derivatives of *S. cerevisiae* SK1 strains. The *HIS4*::*LEU2* locus has been described by Hunter and Kleckner (2001). The genotype and strain details are described in Table S1.

### DNA damage sensitivity assay

Yeast cells were streaked onto YPG plates (1% yeast extract, 2% Bacto peptone, 3% glycerol, and 2% Bacto agar) from the -80°C deep-freezer stock and incubated at 30°C for 18 h. Cells were streaked onto YPD plates (1% yeast extract, 2% Bacto peptone, 2% glucose, and 2% Bacto agar) and incubated at 30°C for 48 h. A single colony was selected and inoculated into 2 ml of YPD liquid medium (1% yeast extract, 2% Bacto peptone, and 2% glucose) at 30°C for 24 h. Saturated cells were serially diluted (10^-1^, 10^-2^, 10^-3^, 10^-4^, and 10^-5^). Cells were spotted onto YPD plates containing the indicated concentrations of DNA-damaging agents and the RFA1-depletion chemical (0.03% MMS, 40 mM HU, 20 μM CuSO_4_, and 2 mM IAA). Plates were incubated at 30°C for 2 days.

### Meiotic time course

The experimental procedure for inducing meiosis was performed as previously described (Cho et al., 2016; Hong et al., 2013, 2019b; Kim et al., 2010; Kong et al., 2017; Rhee et al., 2023; Yoon et al., 2016). Briefly, cells were streaked onto YPG plates from the -80°C freezer and incubated at 30°C for 18 h. Selected cells from the YPG plates were streaked onto YPD plates and incubated at 30°C for 48 h. A single colony was inoculated into 2 ml of YPD liquid medium and grown at 30°C for 24 h. Saturated cultures were diluted into SPS medium (0.5% yeast extract, 1% Bacto peptone, 0.17% yeast nitrogen base without amino acids, 0.5% ammonium sulfate, 1% potassium acetate, and 50 mM potassium biphthalate; pH was adjusted to 5.5 with KOH) and incubated at 30°C for 18 h to synchronize cells in the G_0/1_ phase. Cells were harvested and resuspended in sporulation medium (SPM; 1% potassium acetate, 0.02% raffinose, and 2 drops/L antifoam). For physical DNA analysis, cells were resuspended in 0.1 mg/ml trioxsalen (Sigma, T1637) and exposed to 365 nm UV light for 15 min to induce crosslinking. For analysis of meiotic nuclear division, cells were harvested at each time point and fixed in 40% ethanol and 0.1 M sorbitol. To observe meiotic progression, nuclei were stained with 4',6-Diamidino-2-phenylindole dihydrochloride (DAPI) and the number of nuclei per cell was analyzed by fluorescence microscopy (Olympus BX53). Cells with two nuclei were scored as having completed meiosis I (MI), and four nuclei were scored as having completed meiosis II (MII). At least 200 cells were counted for each time point in each strain.

### Chromosomes spreading and immunofluorescence

Chromosome spreads for immunofluorescence analysis were prepared as previously described (Hong et al., 2019b; Joo et al., 2024; Yoon et al., 2016). Briefly, cells were lysed and fixed on a clean slide using 1% Lipsol and 3% paraformaldehyde containing 3.4% sucrose. Slides were soaked in 0.2% Photo-Flo (Kodak, 146-4510), transferred to TBS buffer (136 mM NaCl, 3 mM KCl, and 25 mM Tris–HCl, pH 8.0), and incubated for 15 min. The following antibodies were used for immunostaining: rabbit polyclonal Rad51 antibody (diluted 1:200); rat polyclonal Dmc1 antibody (diluted 1:200); mouse monoclonal HA antibody (diluted 1:400; Santa Cruz Biotechnology, sc-7392); rabbit polyclonal Zip1 antibody (diluted 1:400; Santa Cruz Biotechnology, sc-33733); mouse monoclonal Flag antibody (diluted 1:400; Santa Cruz Biotechnology, sc-166355); secondary TRITC-conjugated goat anti-rabbit lgG (diluted 1:400; Jackson ImmunoResearch, 111-025-144); secondary Alexa 488-conjugated goat anti-mouse IgG (diluted 1:400; Jackson ImmunoResearch, 115-545-003); secondary FITC-conjugated goat anti-rat IgG (diluted 1:400; Jackson ImmunoResearch, 112-095-003). Images were acquired using a Nikon Eclipse Ti fluorescence microscope with a Nikon DS-Qi2 monochrome camera. Image deconvolution was performed using Nikon NIS software.

### Western blot analysis

Western blot analysis was performed as previously described (Lee et al., 2021; Moon et al., 2022; Yoon et al., 2016). Cells were resuspended in 0.3 M NaOH at room temperature for 5 min and harvested. Cells were resuspended in the sample buffer and heated at 95°C for 5 min. Protein lysates were separated by sodium dodecyl sulfate–polyacrylamide gel electrophoresis (SDS–PAGE) and transferred to polyvinylidene fluoride (PVDF) membranes (Milipore, USA). The following antibodies were used in this study: mouse monoclonal Myc antibody (diluted 1:5000; Santa Cruz Biotechnology, sc-40); rabbit polyclonal Rad51 antibody (diluted 1:5000); rat polyclonal Dmc1 antibody (diluted 1:5000); mouse monoclonal Pgk1 antibody (diluted 1:10,000; Invitrogen, 459250); secondary HRP conjugated goat anti-mouse IgG (diluted 1:10,000; Jackson Immuno Research, 115-035-003); HRP-conjugated goat anti-rabbit IgG (diluted 1:10,000; Jackson Immuno Research, 111-035-003); HRP-conjugated goat anti-rat IgG (diluted 1:10,000; Jackson Immuno Research, 112-035-003). Signals were detected using a ChemiDoc MP imaging system (Bio-Rad).

### DNA analysis

DNA physical analysis was performed as previously described (Cho et al., 2016; Hong et al., 2013; Kim et al., 2010; Kong et al., 2017; Lee et al., 2017; Rhee et al., 2023; Yoon et al., 2016). For one-dimensional (1D) gel analysis, 2 μg of genomic DNA was digested with XhoI at 37°C for 3 h. The resulting DNA species were resolved by electrophoresis in a 0.6% UltraKem LE agarose gel prepared in 1x TBE buffer without ethidium bromide (EtBr) at ~2 V/cm for 24 h. Southern blot hybridization was performed as previously described (Kim et al., 2010; Lee et al., 2021; Rhee et al., 2023).

### Statistical analysis

All statistical analyses were performed using Excel or Prism software (GraphPad) (Lord et al., 2020). All figures representing data are shown as mean values, with error bars (SD), as shown in the figure legends. The number of biological replicates used to represent data was detailed in each figure legend. P values for all relevant comparisons are determined by Student’s *t*-test (ns, p > 0.05; *, p ≤ 0.05; **, p ≤ 0.01; ***, p ≤ 0.001).

## Results

### Rfa1 depletion impairs DSB repair and affects meiotic progression

RPA is a central factor in DNA metabolism, acting at the interface of DNA replication, repair, and checkpoint signaling through the associated high-affinity binding to ssDNA intermediates (Brill and Stillman, 1991; Chen and Wold, 2014; Joo et al., 2024; San Filippo et al., 2008; Symington et al., 2014; Wold, 1997). During S-phase, RPA stabilizes ssDNA exposed at replication forks, whereas in homologous recombination, RPA protects resected DNA ends and facilitates recombinase loading (Brill and Stillman, 1991; Chen and Wold, 2014; Joo et al., 2024; San Filippo et al., 2008; Symington et al., 2014; Wold, 1997). Given these dual roles, perturbation of RPA function is expected to affect DNA replication-associated stress and recombination-dependent DNA repair differentially.

To evaluate the functional impact of Rfa1 depletion, we examined cellular sensitivity to genotoxic agents that impose distinct challenges to DNA metabolism. Thus, cells were exposed to methyl methanesulfonate (MMS) or hydroxyurea (HU), two well-characterized agents that differentially affect DNA replication and repair pathways (Chen et al., 2010; Li and Heyer, 2008). MMS induces alkylation damage that leads to replication fork collapse and DNA DSB formation, lesions that require homologous recombination for efficient repair (Li and Heyer, 2008). In contrast, HU depletes intracellular dNTP pools, resulting in replication fork stalling without directly generating DNA breaks (Li and Heyer, 2008). In YPD medium, the *RFA1*–*AID* strain exhibited growth similar to wild-type (WT) cells, indicating that C-terminal AID–9Myc tagging of Rfa1 does not impair the basal function of RFA1 in the absence of IAA (Fig. 1A and 1B). In contrast, auxin-induced depletion of Rfa1 resulted in high sensitivity to MMS, with markedly reduced viability even at low serial dilutions. This MMS hypersensitivity is consistent with the critical requirement for Rfa1 in recombination-mediated repair of replication-associated DNA damage. However, the growth of the Rfa1-depleted cells on the HU-containing plates was largely comparable to that of the WT (Fig. 1B). This observation suggests that partial replication fork stalling induced by HU can be tolerated under these conditions, or that residual RPA activity is sufficient to support fork stabilization. Collectively, these results indicate that Rfa1 depletion is consistent with a major role in homologous recombination-dependent repair pathways. In contrast, Rfa1 depletion has a limited impact on HU-induced replication stress, suggesting that RPA function may not be strictly required under the conditions tested.

Since Rfa1-depleted cells are sensitive to MMS, we next examined whether Rfa1 depletion affects meiotic progression. To minimize the effects of pre-meiotic DNA replication stress, OsTIR1 expression was induced with 20 μM CuSO_4_ at 2 h, and 2 mM IAA was added at 2.5 h to trigger Rfa1 degradation. Meiotic progression was monitored by scoring nuclear divisions (MI and MII) (Fig. 1C). In the *RFA1*–*AID* [– IAA] condition, meiotic divisions were similar to those in the WT strain. However, the *RFA1*–*AID* [+ 2.5 h IAA] condition exhibited defects in meiotic progression, with an approximately 40% reduction in cells at 24 h (Fig. 1C). This result indicates that Rfa1 is required for repair of programmed DSBs and that Rfa1 depletion is the primary cause of meiotic progression failure. To verify the efficiency of Rfa1 depletion, we performed Western blot analysis using an anti-Myc antibody to detect *RFA1*–*AID*–*9Myc* (Figs. 1D and S1). In the *RFA1*–*AID* [– IAA] condition, Rfa1 protein levels remained stable throughout meiotic prophase. In contrast, in the *RFA1*–*AID* [+ 2.5 h IAA] condition, the Rfa1 protein became undetectable after 2.5 h. These results demonstrate that Rfa1 is rapidly degraded by the AID system following IAA treatment (Fig. 1D).

### Rfa1 depletion impairs recombinase filament formation and fails to complete repair

During meiotic recombination, Dmc1/Rad51-ssDNA filament formation is a critical step that enables efficient homology search, strand invasion, and subsequent repair completion (Bishop and Zickler, 2004; Sansam and Pezza, 2015; Zickler and Kleckner, 1999). RPA plays a central role in the early stages of this process by binding to ssDNA generated at DNA DSBs. By stabilizing ssDNA intermediates, RPA prevents secondary-structure formation and provides a platform for recruiting downstream recombination factors (Brill and Stillman, 1991; Chen and Wold, 2014; San Filippo et al., 2008; Symington et al., 2014; Wold, 1997). The RecA homologs Rad51 and Dmc1 then replace RPA and assemble on ssDNA to form nucleoprotein filaments capable of homology search and strand invasion (Hunter and Kleckner, 2001; Joudeh et al., 2025; San Filippo et al., 2008). Rad51 functions as the main recombinase during mitotic recombination, whereas Dmc1 is a meiosis-specific recombinase that mediates IH strand exchange. During meiosis, Rad51 plays a supporting role in Dmc1-mediated recombination (Da Ines et al., 2022; Hunter and Kleckner, 2001; San Filippo et al., 2008). Although RPA initially occupies ssDNA, RPA is dynamically displaced or reorganized during recombinase loading, suggesting that proper RPA turnover is essential for productive filament formation.

**Fig. 2.**
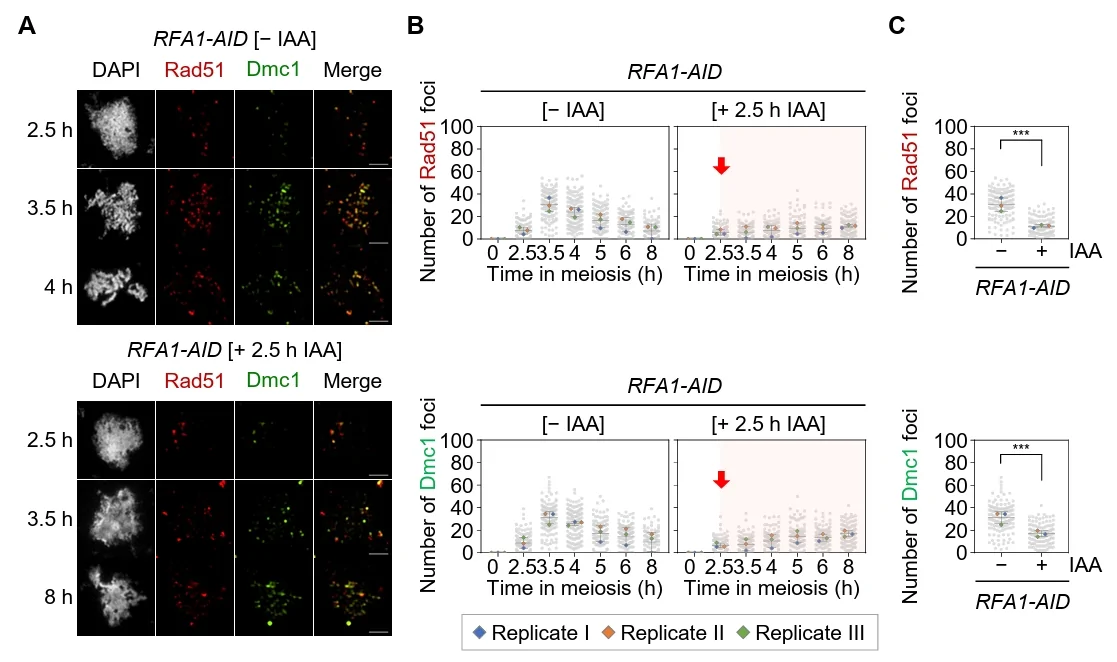
Rfa1 is required for the formation of Rad51 and Dmc1 foci during meiotic recombination. (A) Representative immunofluorescence image of a meiotic nuclear spread from *RFA1*–*AID* strains in the absence [– IAA] or presence [+ IAA] of IAA. Cells were stained with anti-Rad51 (red) and anti-Dmc1 (green). CuSO_4_ (20 μM) was added at 2 h to induce OsTIR1 expression, and IAA (2 mM) was added at 2.5 h to promote Rfa1 degradation. Scale bar = 2.5 μm. (B) Quantification of Rad51 and Dmc1 foci per nucleus in the *RFA1*–*AID* strain during meiotic recombination. Data are presented as the mean ± SD (three independent experiments). (C) Maximum peak time point of Rad51 and Dmc1 foci in the *RFA1*–*AID* strain shown in (B) [– IAA at 3.5 h] and [+ IAA at 8 h]. Data are presented as the mean ± SD (three independent experiments). P values for relevant comparisons are determined by Student’s *t*-test.

To investigate the effect of Rfa1 depletion on recombinase filament assembly, we analyzed the dynamics of Rad51 and Dmc1 focus formation during meiotic recombination (Fig. 2). In the *RFA1*–*AID* [– IAA] condition, Rad51 and Dmc1 foci peaked at 3.5 h after meiotic induction, reaching 30.4 ± 4.92 and 31.19 ± 4.46 (standard deviation, SD), respectively (Fig. 2). Following the peak time point, both Rad51 and Dmc1 foci gradually disappeared, indicating successful strand invasion and resolution of recombination intermediates. In contrast, the numbers of Rad51 and Dmc1 foci in the *RFA1*–*AID* [+ 2.5 h IAA] condition were significantly reduced at 3.5 h (Rad51: 5.96 ± 4.12; Dmc1: 7.23 ± 4.06 (SD)), and the foci persisted at later time points (Fig. 2). Notably, Rfa1 depletion did not alter the expression levels of Rad51 and Dmc1 (Fig. S1). These results suggest that Rfa1 depletion impairs Rad51 and Dmc1 filament formation and disrupts proper turnover of recombination intermediates. The reduction in Rad51 and Dmc1 focus formation, coupled with the prolonged persistence of these proteins at later stages, indicates a defect in presynaptic filament assembly and subsequent repair progression.

### Rfa1 depletion disrupts chromosome axis formation and SC assembly

**Fig. 3.**
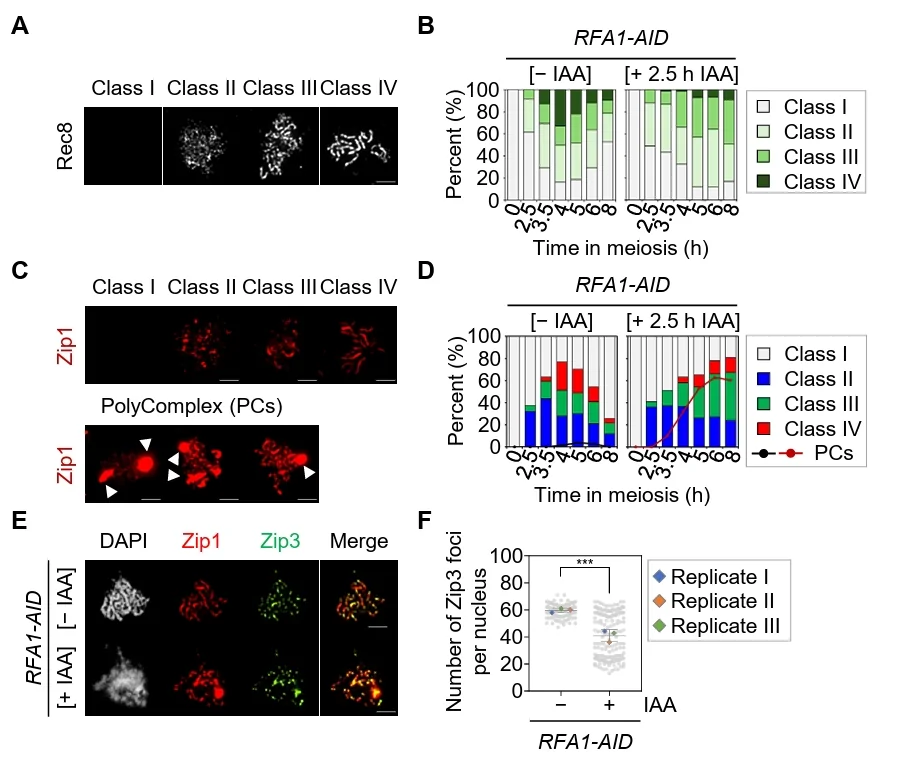
Depletion of Rfa1 causes defects in meiotic recombination progression. (A) Representative immunofluorescence images of meiotic chromosome spreads stained with anti-Rec8 in the *RFA1*–*AID* strain. CuSO_4_ (20 μM) was added at 2 h to induce OsTIR1 expression, and IAA (2 mM) was added at 2.5 h to induce Rfa1 degradation. Nuclear spreads were classified into four categories based on the Rec8 staining pattern: Class I, no staining; Class II, a modest number of chromosomes; Class III, extended or short linear chromosomes; Class IV, a full array of chromosomes. Scale bar = 2.5 μm. (B) Quantification of Rec8 staining classes at each time point shown in (A). (C) Representative images of meiotic chromosome spreads stained with anti-Zip1 in the *RFA1*–*AID* strain. CuSO_4_ and IAA were added as in (A). Nuclear spreads were classified into four categories based on the Zip1 staining pattern: Class I, blank; Class II, dotty chromosomes; Class III, short or discontinuous linear chromosomes; Class IV, full-array chromosomes. White arrowheads indicate polycomplexes (PCs). Scale bar = 2.5 μm. (D) Quantification of Zip1 staining classes at each time point shown in (C). The line graph indicates the percentage of cells with PCs. (E) Representative meiotic chromosome spreads from *RFA1*–*AID* strains immunostained with anti-Zip1 (red) and anti-Flag (for Zip3 staining, green). CuSO_4_ (20 μM) was added at 2 h to induce OsTIR1 expression, and IAA (2 mM) was added at 2.5 h to induce Rfa1 degradation. Scale bar = 2.5 μm. (F) Quantification of Zip3 foci per nucleus at the peak time points of Zip3 expression during meiotic recombination: [– IAA] at 4 h and [+ IAA] at 6 h. Data are presented as the mean ± SD (three independent experiments). P values for relevant comparisons are determined by Student’s *t*-test.

Meiotic recombination occurs within a highly organized chromosome axis structure that is established during early prophase I (Blat et al., 2002; Borde and de Massy, 2013). The chromosome axis is composed of cohesion complexes containing the meiosis-specific subunit Rec8, together with axis-associated proteins such as Red1 and Hop1, which provide a structural framework that organizes chromatin into loop–axis arrays (Hollingsworth and Byers, 1989; Rockmill and Roeder, 1990; Shodhan et al., 2022; Smith and Roeder, 1997). This axis supports homolog pairing and spatially coordinates the initiation of meiotic recombination. Meanwhile, the SC assembly is tightly coupled to chromosome axis formation (Hong et al., 2019a; Keeney et al., 2014; Zickler and Kleckner, 2015). During zygotene, the transverse filament protein Zip1 polymerizes between aligned homologous chromosome axes to form the central region of the SC, thereby stabilizing homologous interactions and facilitating CO formation (Börner et al., 2004; Cesar and Kim, 2026; Gold and Kim, 2026; Jo et al., 2022; Page and Hawley, 2004; Sym et al., 1993; Zickler and Kleckner, 1999). Proper loading and distribution of Rec8 along chromosome axes are essential for Zip1 assembly, as the axis serves as a scaffold for SC formation (Page and Hawley, 2004; Sym et al., 1993; Zickler and Kleckner, 1999). To confirm the progression of structural formation, we examined the chromosome axis formation and SC assembly in the *RFA1*–*AID* strain. Thus, chromosomes and proteins were stained with anti-HA (to detect Rec8), and chromosome spreads were stained with anti-Zip1 to monitor axis formation and SC assembly (Fig. 3A–3D). Rec8 patterns were categorized into four categories: Class I (no staining), Class II (foci on a modest number of chromosomes), Class III (extended or short linear chromosomes), and Class IV (full-array chromosome) (Fig. 3A and 3B). Zip1 assembly was similarly classified into four categories: Class I (blank), Class II (dotted chromosomes), Class III (short or discontinuous linear chromosomes), and Class IV (full-array chromosomes) (Fig. 3C and 3D). In the *RFA1*–*AID* [– IAA] condition, Rec8 formed continuous axial structures, and Zip1 assembled into extended linear stretches closely aligned with these axes. Both the axis and SC signals peaked at 4 h and then gradually declined, accompanied by a reduction in full-array chromosomes. These results indicate that homologous chromosomes become fully synapsed and that recombination repair is completed (Fig. 3A–3D), reflecting normal SC assembly during mid-prophase I. In contrast, the *RFA1*–*AID* [+ 2.5 h] condition exhibited defects in both chromosome axis formation and SC assembly. In Rfa1-depleted cells, the proportion of full-axis chromosomes was markedly reduced relative to the *RFA1*–*AID* [– IAA] condition. Most cells appeared fragmented and disorganized, indicating impaired axis integrity (Fig. 3A and 3B). Consistent with these axis defects, Zip1 failed to assemble into continuous linear structures and instead exhibited discontinuous localization along chromosomes. Moreover, Zip1 accumulated polycomplexes (PCs) that did not associate with chromosomes; these aggregates were present in approximately 62.6% of cells (Fig. 3C and 3D). The formation of these PCs suggests a failure in the nucleation or stabilization of SC assembly along chromosome axes (Chua and Roeder, 1998; Sym and Roeder, 1995). Together, these results demonstrate that Rfa1 is required for proper coordination between recombination progression and chromosome synapsis.

### Rfa1 is required for proper CO designation via the ZMM pathway

The SC promotes chromosome synapsis and CO designation events during meiotic recombination (Cesar and Kim, 2026; Machovina et al., 2016; Nadarajan et al., 2017; Pattabiraman et al., 2017). In particular, because the SC is intimately associated with the formation and stabilization of ZMM-dependent crossover intermediates, defects in SC assembly are expected to impair both the efficiency and stability of crossover designation (Cesar and Kim, 2026; Machovina et al., 2016; Nadarajan et al., 2017; Pattabiraman et al., 2017). Therefore, we quantitatively assessed whether CO formation and designation are affected under Rfa1 depletion.

Zip3 is a SUMO E3 ligase and a core ZMM complex component that recruits synapsis initiation complexes (SICs; Zip2–4 and Spo16) and acts as a primary, early-stage marker of CO designation during meiotic recombination (Agarwal and Roeder, 2000; Voelkel-Meiman et al., 2019). To determine whether Rfa1 depletion affects CO designation during meiotic progression, we quantified Zip3 foci at the pachytene stage (Fig. 3E and 3F). Zip3–Flag foci were stained with anti-Flag and visualized along the SC of surface-spread pachytene chromosomes. In the *RFA1*–*AID* [– IAA] condition, Zip3 foci accumulated efficiently, peaking at 4 h (59.73 ± 1.28 foci (SD)) (Fig. 3E and 3F), similar to the WT strain. In contrast, the *RFA1*–*AID* [+ 2.5 h IAA] condition exhibited a 31% reduction relative to *RFA1*–*AID* [– IAA] (41.13 ± 3.58 foci (SD)) (Fig. 3E and 3F). Additionally, the peak of Zip3 accumulation was delayed to 6 h. The reduced number and delayed emergence of Zip3 foci suggest a defect in the establishment or stabilization of CO-designation intermediates upon Rfa1 depletion (Fig. 3E and 3F). These results suggest that although recombination is initiated, progression to ZMM-stabilized CO intermediates is inefficient. The temporal delay in Zip3 foci turnover further suggests that prolonged persistence of recombination intermediates is consistent with impaired repair progression.

### RPA involves the Rad51-dependent pathway of meiosis

**Fig. 4.**
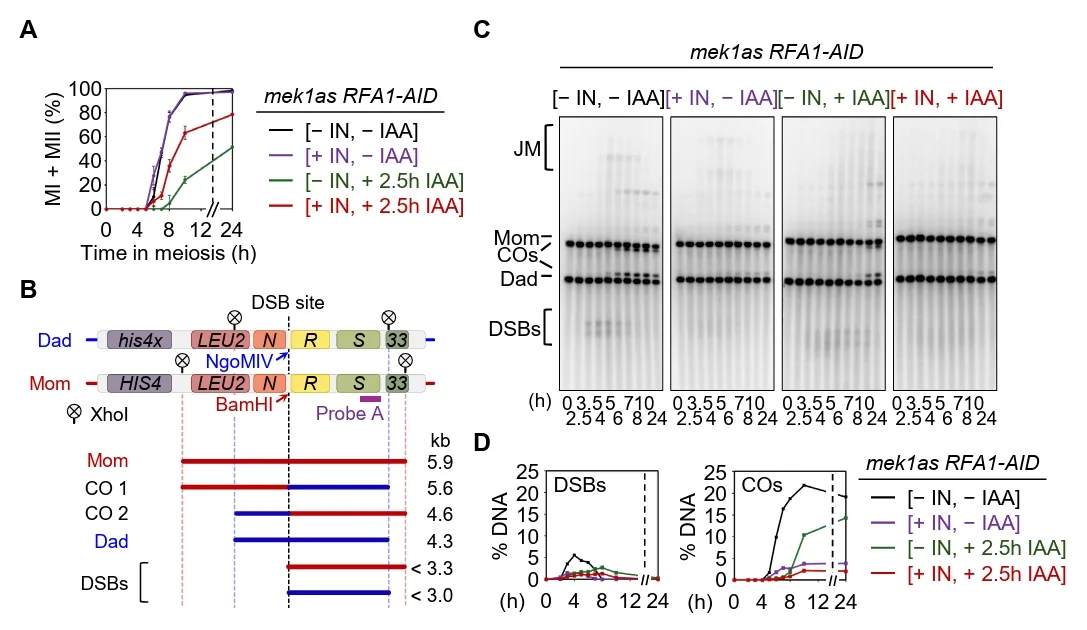
Rfa1 is essential for meiotic DSB and crossover formation. (A) Meiotic nuclear division in the *mek1as* *RFA1*–*AID* strain. Mek1 kinase activity was inhibited by the addition of 1-NA-PP1 (IN; 2 µM) at 0 h. CuSO_4_ (20 μM) was added at 2 h to induce OsTIR1 expression, and IAA (2 mM) was added at 2.5 h to induce degradation of Rfa1. Data are presented as the mean ± SD (N > 200 cells per time point; three independent biological replicates). (B) Schematic of the *HIS4*::*LEU2* recombination hotspot, which generates double-strand breaks (DSBs) during meiosis. Positions of XhoI restriction sites are indicated by an X. Probe A (purple), used for Southern blot analysis, is shown. DSB, double-strand break; CO, crossover. (C) Representative one-dimensional gel images from *mek1as* *RFA1*–*AID* strains. (D) Quantification of DSB and CO levels shown in (C).

Previous studies have suggested that Rfa1 depletion impairs the progression of meiotic DSB repair and homologous recombination-mediated CO formation in the homologous bias pathway (Joo et al., 2024). Therefore, we investigated whether Rfa1 depletion also affects the progression of Rad51-dependent recombination. The *mek1as* allele encodes a kinase with an enlarged ATP-binding pocket, allowing specific inhibition of *mek1as* by the 1-NA-PP1 (IN) inhibitor. Inhibition of Mek1 kinase activity blocks the establishment of homolog bias, causing a rapid switch to sister-biased recombination (Bishop et al., 2001; Kim et al., 2010; Niu et al., 2005; Wan et al., 2004). Thus, we created a *mek1as* *RFA1*–*AID* strain and monitored meiotic recombination at the well-characterized *HIS4*::*LEU2* hotspot on chromosome III using 1D Southern blot-based physical assays (Fig. 4; Hunter and Kleckner, 2001). In the *mek1as* *RFA1*–*AID* [– IN, – IAA] condition, DSBs appeared 2.5 h after meiotic induction and peaked at 4 h. COs were detected shortly after DSB formation and accumulated to high levels at later time points, consistent with normal meiotic recombination (Fig. 4C and 4D; Kim et al., 2010). In the *mek1as* *RFA1*–*AID* [+ IN, – IAA] condition, inhibition of the Mek1 kinase resulted in hyper-resection and reduced DSB levels compared with the *mek1as* *RFA1*–*AID* [– IN, – IAA] condition. Consistently, CO formation was markedly reduced upon Mek1 inhibition: maximal CO levels decreased by approximately 82.6% relative to the control, reaching 21.8% at 10 h in the [– IN, – IAA] condition and 3.8% at 24 h in the [+ IN, – IAA] condition (Fig. 4C and 4D). Mechanistically, Mek1 inhibition accelerates meiotic division by promoting rapid intersister DSB repair, thereby preventing the accumulation of IH recombination intermediates required to sustain pachytene checkpoint signaling. This premature loss of checkpoint signaling triggers early exit from meiotic prophase and earlier progression through MI and MII (Fig. 4). In contrast, in the *mek1as* *RFA1*–*AID* [– IN, + IAA] condition, low levels of DSBs were detected at early time points, with delayed accumulation peaking at approximately 8 h. CO formation was similarly delayed, initiating around 6 h; maximal CO levels were reduced, reaching 14.29% at 24 h (Fig. 4C and 4D). These results indicate that Rfa1 is required for efficient DSB accumulation and processing, as well as for proper processing of recombination intermediates during meiosis. Importantly, in the *mek1as* *RFA1*–*AID* [+ IN, + IAA] condition, although meiotic division was partially restored, reaching 90% at 24 h compared with 69.9% in the *mek1as* *RFA1*–*AID* [– IN, + IAA] condition, recombination progression was not rescued (Fig. 4). Under these conditions, DSB levels were reduced and delayed, and CO levels were further decreased relative to the *mek1as* *RFA1*–*AID* [+ IN, – IAA] condition, reaching 3.8% at 24 h in the [+ IN, – IAA] condition and 2.1% at 10 h in the [+ IN, + IAA] condition (Fig. 4C and 4D).

### Rfa1 is required for focus turnover of Rad51 and Dmc1

**Fig. 5.**
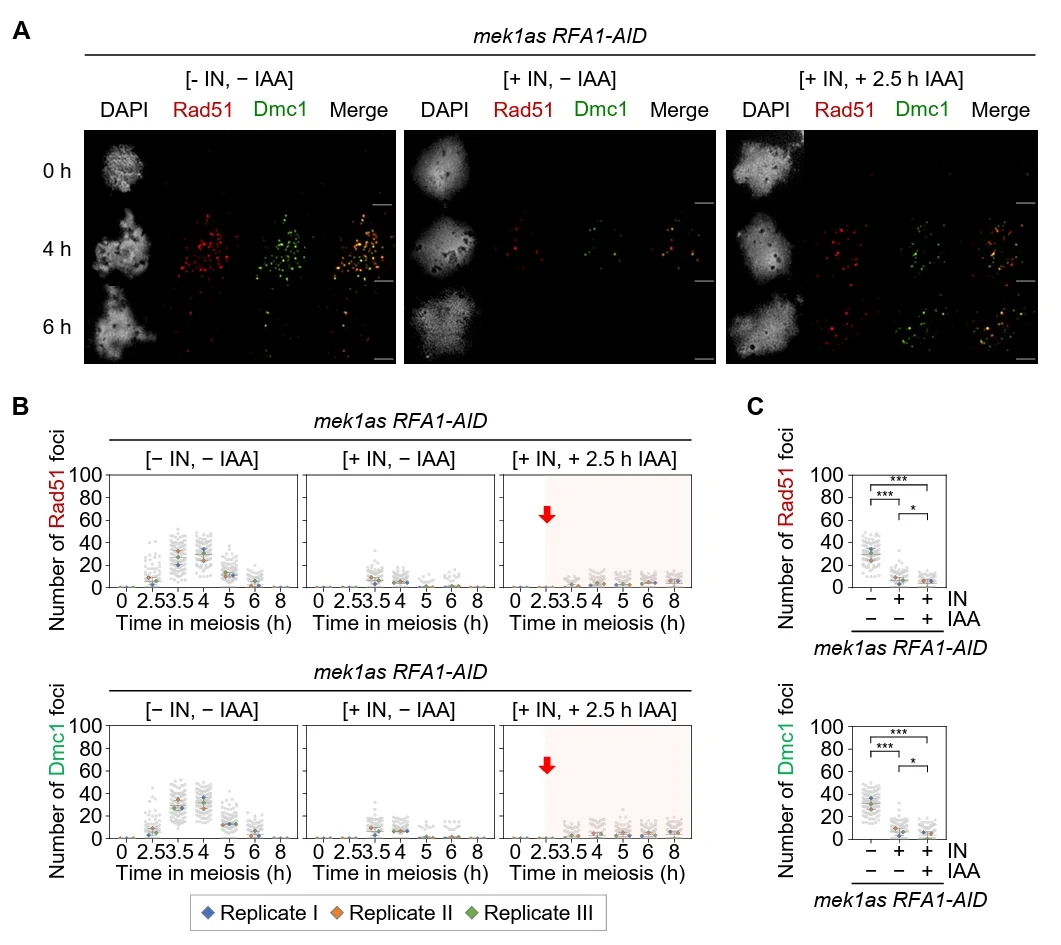
Rfa1 is required for Rad51 and Dmc1 foci formation. (A) Representative images of meiotic chromosome spreads from the *mek1as* *RFA1*–*AID* strain, immuno-stained with anti-Rad51 (red) and anti-Dmc1 (green). Mek1 kinase activity was inhibited by the addition of 1-NA-PP1 (IN; 2 µM) at 0 h. CuSO_4_ (20 μM) was added at 2 h to induce OsTIR1 expression, and IAA (2 mM) was added at 2.5 h to induce Rfa1 degradation. Scale bar = 2.5 µM. (B) Quantification of Rad51 and Dmc1 foci per nucleus in the *mek1as* *RFA1*–*AID* strain. Data are presented as the mean ± SD (three independent experiments). (C) Maximum peak time point of Rad51 and Dmc1 foci in the *mek1as* *RFA1*–*AID* strain shown in (B) [– IN, – IAA at 4 h], [+ IN, – IAA at 3.5 h] and [+ IN, + IAA at 8 h]. Data are presented as the mean ± SD (three independent experiments). P values for relevant comparisons are determined by Student’s *t*-test.

We analyzed Rad51 and Dmc1 foci dynamics to determine whether Rfa1 is required for recombination repair independently of partner choice in the *mek1as* *RFA1*–*AID* strain during meiotic recombination (Fig. 5). In the *mek1as* *RFA1*–*AID* [+ IN, – IAA] condition, a small number of Rad51 and Dmc1 foci were observed, reaching 4.9 ± 0.6 and 6.6 ± 0.1 (SD), respectively at 4 h, and disassembling rapidly by 5 h. This result shows that Rad51 and Dmc1 foci are efficiently resolved by approximately 5 h after meiotic induction (Fig. 5). This timely disappearance of recombinase foci is consistent with efficient intersister repair when homolog bias is abolished, and lesions are rapidly repaired via the intersister pathway. In contrast, the *mek1as* *RFA1*–*AID* [+ IN, + IAA] condition shows persistent Rad51 and Dmc1 foci during meiotic recombination (Fig. 5). These results indicate that Rfa1 depletion disrupts the Rad51-dependent meiotic recombination repair pathway. Together with our physical and cytological analyses, these data demonstrate that Rfa1 is required for the completion of meiotic recombination.

## Discussion

Our results demonstrate that Rfa1 is a central factor required for efficient progression of yeast meiotic recombination, coordinating recombination intermediate dynamics with chromosome organization and crossover formation. This study used an AID system to deplete Rfa1 during meiotic prophase I of *S. cerevisiae*, thereby minimizing indirect effects associated with defects in pre-meiotic DNA replication. Thus, this approach enabled us to examine the role of Rfa1 directly in meiotic recombination and prophase-specific events of yeast meiosis.

### Rfa1 is required for recombination-dependent DNA repair during meiosis

We first assessed the impact of Rfa1 depletion on cellular responses to two distinct genotoxic stresses, MMS and HU. Rfa1 depletion rendered cells hypersensitive to MMS, whereas cellular sensitivity to HU was comparatively modest. Since MMS-induced lesions frequently generate recombination-dependent DNA repair intermediates, whereas HU primarily causes replication fork stalling, this differential sensitivity implies a lower quantitative threshold for Rfa1 at stalled fork junctions than the high-density loading required for DNA resection and homology search (Li and Heyer, 2008; Xiao et al., 1996). These observations are consistent with the idea that RPA is differentially required in replication-associated stress and recombination-dependent repair. HU primarily induces replication forks stalling that activates checkpoint signaling rather than generating extensive DNA damage requiring homologous recombination. Previous studies have shown that a relatively low level of RPA is sufficient for Mec1-Ddc2 checkpoint activation (Chen et al., 2013; Sampathkumar et al., 2024), suggesting that residual RPA activity in the *RFA1*–*AID* strain may be sufficient to stabilize stalled replication forks and support checkpoint signaling under HU conditions.

### Rfa1 is required for efficient recombinase-mediated recombination progression

We analyzed the dynamics of Rad51 and Dmc1 during meiotic recombination in Rfa1-depleted cells and observed both a reduction in early focus formation and a prolonged persistence of these foci at later stages. The significant reduction in early focus formation indicates that Rfa1-mediated ssDNA coating is a critical prerequisite for the initial loading of Rad51 and Dmc1 at DSB sites. During meiotic recombination, RPA must efficiently coat ssDNA to eliminate secondary structures, thereby generating an appropriate substrate for the recruitment and stabilization of Rad51/Dmc1 filaments by specialized recombination mediators (Joo et al., 2024; New et al., 1998; Sung, 1997). Consequently, reduced Rfa1 levels likely impair the transition from resected DNA ends to stable nucleoprotein filaments. Previous studies have demonstrated that RPA plays a critical role in recombinase loading rather than acting solely as a passive ssDNA-binding factor. RPA facilitates the recruitment of Rad52, which mediates Rad51 loading onto ssDNA, and depletion of RPA results in a marked reduction in Rad51 foci formation despite the potential presence of ssDNA (Chen et al., 2013; Joo et al., 2024). These findings suggest that recombinase loading can be impaired even when ssDNA is generated. Furthermore, the aberrant persistence of the remaining recombinase foci at later stages suggests a defect in the maturation or turnover of recombination intermediates (Joo et al., 2024). Collectively, these results demonstrate that Rfa1 is required for efficient loading of Rad51 and Dmc1 and for stabilizing productive intermediates, thereby ensuring the correct progression of meiotic recombination.

### Depletion of Rfa1 impairs chromosome axis formation and synapsis assembly, leading to defective ZMM-dependent CO designation

The disrupted Rec8 axis structures, discontinuous Zip1 localization, and extensive Zip1 PC formation in Rfa1-depleted cells suggest a failure to coordinate recombination intermediates with chromosome morphogenesis (Keeney et al., 2014; Zickler and Kleckner, 2015). Specifically, defects in chromosome axis and SC formation in Rfa1-depleted cells suggest that Rfa1-mediated ssDNA stabilization during meiotic recombination contributes to proper chromosome axis assembly. In addition, RPA regulates Mec1-dependent checkpoint signaling that controls Hop1 phosphorylation and Mek1 activation (Sampathkumar et al., 2024), suggesting that defects in recombination pathway may affect chromosome axis organization. The structural defects in the chromosome axis directly impair SC assembly, as shown by the inability of Zip1 to form continuous linear structures and the aggregation of Zip1 PCs. Together, these results suggest that chromosome axis and SC defects arise indirectly from defects in recombination and checkpoint signaling. Furthermore, we found that these structural defects were accompanied by impaired ZMM-dependent CO designation. ZMM proteins, such as Zip3, preferentially associate with stabilized recombination intermediates that mature into class I COs and serve as nucleation sites for SC assembly (Agarwal and Roeder, 2000; Voelkel-Meiman et al., 2019). Consistent with defects in SC assembly, Rfa1 depletion led to a significant reduction and delay in Zip3 focus formation, indicating impaired CO designation via the ZMM pathway. In the Rfa1-depleted strain, fewer Zip3 foci and a delay in accumulation suggest that recombination intermediates formed in the absence of Rfa1 fail to achieve a stable configuration competent for ZMM recruitment. Thus, Rfa1 is required to couple recombination intermediate maturation with chromosome axis and SC assembly, thereby enabling efficient ZMM recruitment and Class I CO designation.

### Rfa1 functions in the Rad51-dependent repair pathway during meiosis

A key finding of our study is that recombination defects caused by Rfa1 depletion are not rescued by activating the Rad51-dependent repair pathway. In budding yeast, inhibition of Mek1 kinase activity abolishes homolog bias and promotes Rad51-mediated sister chromatid repair (Kim et al., 2010; Niu et al., 2005). However, even when Mek1 inhibition favors intersister DSB repair, Rfa1 depletion still impairs recombination, as revealed by both physical and cytological analyses. Under conditions of Rfa1 depletion combined with Mek1 inhibition, DSB processing remains inefficient, recombinase foci persist abnormally, and CO formation is severely compromised, despite the availability of the sister chromatid as a repair template. These results indicate that the persistence of Rad51 and Dmc1 foci reflects a defect in the maturation of recombination intermediates. Specifically, Rfa1 depletion disrupts ssDNA structural integrity, resulting in unstable recombination intermediates that fail to support productive repair, regardless of whether a homolog or a sister chromatid serves as the template. Indeed, Rfa1 depletion disrupted the dynamics of recombination intermediates, chromosome axis formation, SC assembly, and ZMM-dependent CO designation during meiosis. Therefore, we conclude that Rfa1 is fundamentally required for the progression and completion of meiosis.

## Supplementary Information

Table S1. List of *S. cerevisiae* used in this study

Fig. S1. Protein expression analysis in the *RFA1*–*AID* strain during meiosis. (A) Expression of Rad51, Dmc1, and Pgk1 proteins in the *RFA1*–*AID* strain during meiotic recombination. CuSO_4_ (20 μM) was added at 2 h to induce OsTIR1 expression, and IAA (2 mM) was added at 2.5 h to induce Rfa1 degradation. Protein extracts were analyzed by Western blotting with antibodies against Rad51, Dmc1, and Pgk1. Asterisks indicate non-specific bands. (B) Unprocessed Western blot images corresponding to Fig. 1D. (C) Unprocessed Western blot images corresponding to Fig. S1A.
