# Supplementary material for: Prophase roles of replication protein A in crossover formation and meiotic progression
Source: J Microbiol. 2026 Jun 18;64(6):e2604001. doi: 10.71150/jm.2604001 (PMC13577082; doi:10.71150/jm.2604001)
Supplement: Supplementary file 1 [file jm-2604001-Supplementary-Fig-S1.pdf]

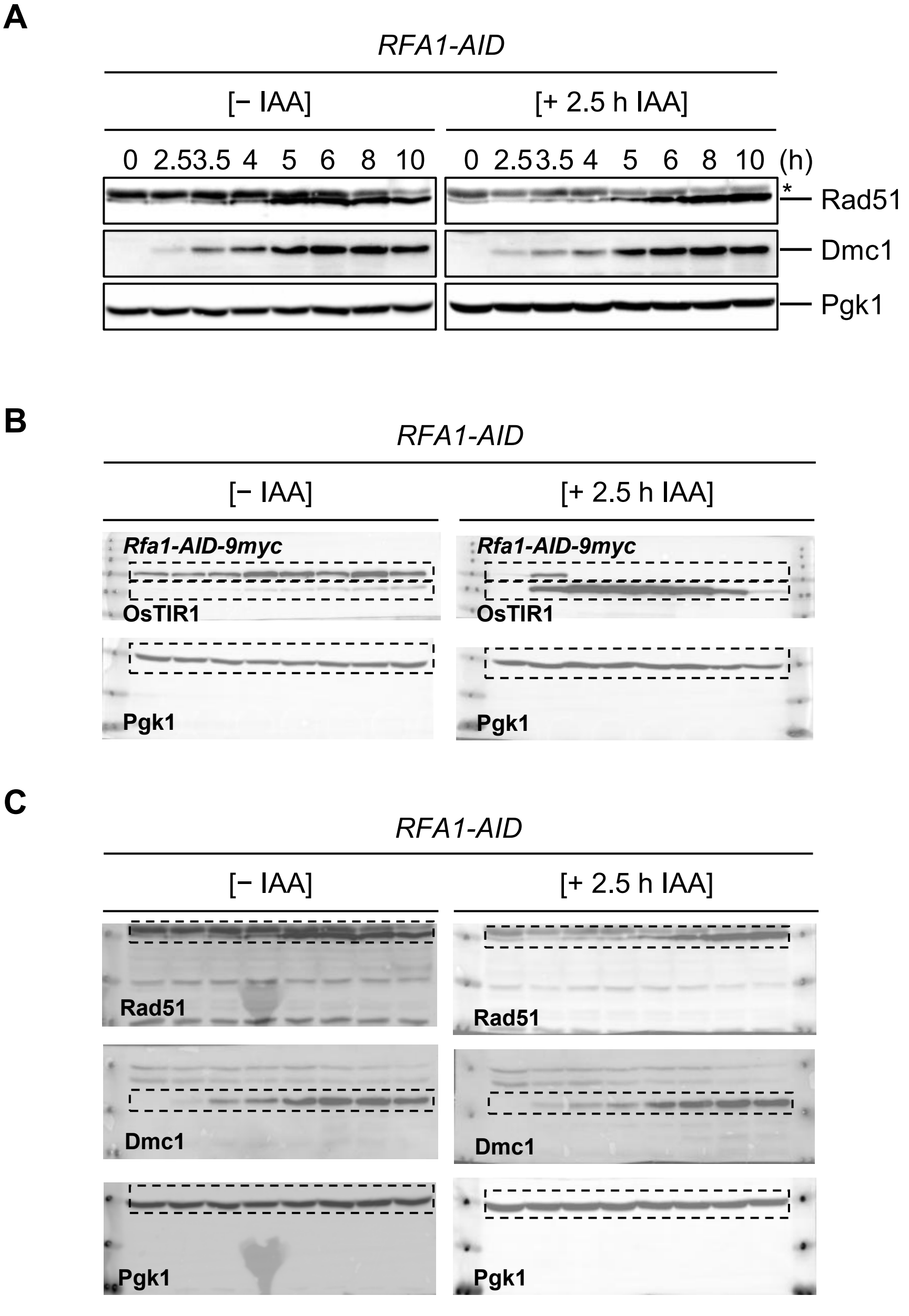

Fig. S1. Protein expression analysis in the *RFA1–AID* strain during meiosis. (A) Expression of Rad51, Dmc1, and Pgk1 proteins in the *RFA1–AID* strain during meiotic recombination. $CuSO_4$ (20 μM) was added at 2 h to induce OsTIR1 expression, and IAA (2 mM) was added at 2.5 h to induce Rfa1 degradation. Protein extracts were analyzed by Western blotting with antibodies against Rad51, Dmc1, and Pgk1. Asterisks indicate non-specific bands. (B) Unprocessed Western blot images corresponding to Fig. 1D. (C) Unprocessed Western blot images corresponding to Fig. S1A.
