# Supplementary material for: Prophase roles of replication protein A in crossover formation and meiotic progression
Source: J Microbiol. 2026 Jun 18;64(6):e2604001. doi: 10.71150/jm.2604001 (PMC13577082; doi:10.71150/jm.2604001)
Supplement: Supplementary file 2 [file jm-2604001-Supplementary-Table-S1.pdf]

Table S1. List of *S. cerevisiae* used in this study

Strain†	Genotype‡
KKY6439	*MATa*/*MATα*, *REC8-3HA::URA3*/REC8, Rfa1-AID-9myc-HygB/”, pCUP1-KanMX-OsTIR1-9myc-URA3/”
KKY6660	*MATa*/*MATα*, *ZIP3-3FLAG::KanMX*/ZIP3, Rfa1-AID-9myc-HygB/”, pCUP1-KanMX-OsTIR1-9myc-URA3/”
KKY6680	*MATa/MATα, mek1::LEU2::mek1-as1::URA3*/”, *REC8-3HA::URA3*/REC8, Rfa1-AID-9myc-HygB/”, pCUP1-KanMX-OsTIR1-9myc-URA3/”
KKY6944	*MATa/MATα*, *HIS4::LEU2-(BamHI)/his4x::LEU2-(NgoMIV)-URA3*, *mek1::LEU2::mek1-as1::URA3*/”, *Rfa1-AID-9myc-HygB*/”, *pCUP1-KanMX-OsTIR1-9myc-URA3/”*

†All strains are isogenic derivatives of SK1 background.

‡All strains are also homozygous for the mutation *ho::hisG*, *leu2::hisG*, *ura3 (ΔPstI-SmaI)*.
